# Evaluation of Obesity Management Recorded in Electronic Clinical History: A Cohort Study

**DOI:** 10.3390/jcm9082345

**Published:** 2020-07-23

**Authors:** Nuria Trujillo-Garrido, María Ángeles Bernal-Jiménez, María J. Santi-Cano

**Affiliations:** 1 Faculty of Nursing, University of Cádiz, 11207 Cádiz, Spain; nuria.trujillo@uca.es; 2 Institute of Biomedical Research and Innovation of Cádiz (INiBICA), 11003 Cádiz, Spain; m.angeles.bernal@inibica.es; 3 Faculty of Nursing and Physiotherapy, University of Cádiz, 11009 Cádiz, Spain; 4 Research Group on Nutrition: Molecular, Pathophysiological and Social Issues, University of Cádiz, 11009 Cádiz, Spain

**Keywords:** obesity, clinical guidelines, primary care, evidence-based practice

## Abstract

Background: The prevalence of obesity is increasing worldwide. Because of their close proximity to the population, primary care physicians and nurses are in a unique position to motivate and advise patients with obesity on a healthy diet and increased physical activity. Drawing from information recorded in electronic clinical records, we evaluated how the general recommendations included in obesity guidelines are being implemented in routine clinical practice. Methods: This study drew from the following data from a cohort of 209 patients with obesity that attended primary care consultations: electronic clinical records, body mass index (BMI), waist circumference (WC), cardiovascular risk factors, comorbidities and whether their health professional documented compliance with the recommendations of the evidence-based obesity guidelines in their electronic history. Results: Only 25.4% of the clinical records met all the criteria established in the therapeutic guidelines regarding diet prescription, 1.4% for physical activity and 1.5% for behavioral change activities. The patients whose records mentioned diet prescription and physical activity and who received follow-up consultations for both factors had lower average BMI and WC, although this relationship was not significant after adjusting for baseline. Conclusions: We found that only a small number of records in the electronic clinical histories followed the evidence-based obesity guidelines. Recording dietetic prescription and physical exercise in the patient’s clinical record is associated with better control of obesity.

## 1. Introduction

The prevalence of obesity is increasing among people of all ages in high, middle and low- income countries. Obesity raises the risk of several conditions, including type 2 diabetes mellitus (T2DM), hypertension, dyslipidemia, cardiovascular disease, osteoarthritis, sleep apnea, and some cancers [1]. It also constitutes a significant burden on health services [2].

In European countries, the current prevalence of obesity in adults is high (28.1% in England and 23.8% in Spain [3]). The prevalence of obesity in the USA is even higher than in Europe, at 36% [4].

To stop this pandemic, effective strategies need to be developed and applied to both prevent and treat obesity. Obesity should be treated as a chronic disease and its management performed by trained primary care professionals [5].

The traditional treatment of obesity, based only on prescribing increased physical activity and decreased calorie intake, has not been successful in large number of individuals [6,7]. Managing the psychological aspects of the disease and the use of behavioral techniques to improve lifestyle are considered key. However, a number of barriers hinder the effective implementation of comprehensive lifestyle programs for the treatment of patients with obesity, such as limited consultation time, a lack of confidence that obesity advice is effective, a shortage of appropriate clinical space and human resources, such as dietitians and psychologists, and insufficient training of health professionals in behavioral counselling [5]. The lack of efficiency of these therapeutic approaches may also be due to an insufficient understanding of the etiology of obesity [8]. Moreover, individuals with obesity are often perceived as lacking will power and self-discipline and, although current scientific knowledge regarding mechanisms of body weight regulation does not support that view, it could affect the quality of health care provided to people with obesity [9].

The current clinical practice guidelines on obesity recommend carrying out a series of interventions to improve diet and the level of physical activity through behavioral change techniques [10,11]. The European Practical and Patient-Centred Guidelines for Adult Obesity Management in Primary Care recommends the following approaches to obesity treatment (Box 1) [10]:

Box 1 **Obesity management guidelines specifically tailored to General Practitioners (GPs).**

1. Improve communication and motivation is essential for adherence to treatment.
2. Avoid stigmatization in a health care setting.
3. Measure waist circumference.
4. Treat comorbidities as a priority to decrease mortality.
5. Use a multidisciplinary team: GPs, nurses, dietitians, psychologist.
6. Assess weight loss: 5–10% weight loss from initial weight is already sufficient to decreases comorbidities.
7. Consider lifestyle behavior change: Nutrition and physical activity. It will also help to improve body image, self-esteem and quality of life.
8. Avoid weight cycling: If the patient gains 3–4 kg quickly, he/she should not wait too long before visiting the GPs to be assessed.

Primary care professionals (doctors and nurses) play a key role in advising and motivating patients affected by obesity on the health benefits of weight loss. However, despite the obesity epidemic, scientific evidence shows that we are not adequately addressing overweight and obesity. The intensive behavioral counseling for patients with obesity requires regular and systematic documentation in electronic clinical histories for monitoring compliance with the prescription, weight loss, and response to treatment [12]. Health professional–patient engagement is key in the management of obesity. In any routine consultation, a doctor or nurse should weigh the patient and if he/she has obesity they should give an appointment for a consultation with more time to address obesity.

Different studies have evaluated the effectiveness of primary care interventions in treating obesity or how patients with obesity are really managed in primary care [13,14,15,16]; however, these studies have not addressed how evidence-based guidelines are implemented in routine clinical practice. To achieve weight loss, these guidelines recommend a comprehensive approach: diet, physical activity, and behavior therapy. Our hypothesis was that a low number of treatment prescription recorded in the electronic clinical history would follow the evidence-based obesity guidelines and that this is associated with a limited control of obesity, which is evidenced by higher patient adiposity.

This study used information recorded in electronic clinical history to examine how the general recommendations included in the evidence-based obesity guidelines are implemented in routine clinical practice, and to analyze changes in the body mass index and clinical status (in terms of the prevalence of cardiovascular risk factors, diabetes mellitus and major comorbidities associated with obesity, such as acute myocardial infarction and obstructive sleep apnea syndrome) of patients with obesity over a 5-years period (current vs. 5 year before) in a primary care setting.

## 2. Methods

This study drew from data from a cohort of 209 patients with obesity who attended consultations at a primary care center in Guadalajara, Spain. The sample was selected from the Spanish Public Health Service’s electronic health records and the inclusion criteria were as follows: adults over 18 years old diagnosed with obesity (defined as BMI ≥ 30 kg/m^2^) recorded in their electronic clinical record from at least 5 years prior to our study. Exclusion criteria were: obesity secondary to genetic syndromes; hypothalamic or hormonal alterations; any hepatic, cardiac or renal disease that causes body edema; terminal illness; pregnancy or lactation; cognitive deterioration; patient absenteeism from primary care consultations for over a year; and institutionalized patients. Taking into account a population of 510 people affected by obesity in the 2016 period and assuming a 20%, estimated proportion of weight management intervention [17], the required sample size to achieve 4% precision and a confidence level of 90%, was calculated at 177 patients. We contacted all eligible participants, and 209 subjects who met the inclusion criteria decided to participate.

We obtained our data from primary care consultations prospectively, patient interviews and electronic clinical records. During the consultations, we asked the patients to obtain the following data: demographic data; personal history; body mass index (BMI) calculated as (weight (kg)/height^2^ (m)); weight, measured without shoes or heavy clothing and, using standard calibrated scales (Seca 711, Hamburg, Germany) to the nearest 0.1 kg, height, measured using a portable stadiometer (Seca 264 height rod) to the nearest 0.1 cm; waist circumference (WC); cardiovascular risk factors such as smoking; hypertension (systolic blood pressure > 140 mmHg or diastolic blood pressure > 90 mmHg, and/or use of antihypertensive agents); hypercholesterolemia (total plasma cholesterol ≥ 190 mg/dL -5 mmol/L-) and/or use of lipid-lowering medication); hypertriglyceridemia (plasma triglycerides ≥ 150 mg/dL -1.7 mmol/L-) [18] or type 2 diabetes mellitus (T2DM, fasting plasma glucose level ≥ 126 mg/dL -6.99 mmol/l-) and/or use of antidiabetic drugs [19]; major comorbidities associated to obesity (acute myocardial infarction—AMI—and obstructive sleep apnea syndrome—OSAS); the type of treatment prescribed for obesity; patient adherence to treatment (diet, physical exercise and behavioral change, based on closed-ended question: yes or no). The data obtained retrospectively from the electronic clinical record included whether or not a pharmacological treatment for obesity had been prescribed. BMI and WC were recorded in the annual review during the five years prior to our study, as well as underlying cardiovascular risk factors, and major comorbidities (AMI and OSAS) suffered by patients over the five years prior to the time of our study.

The study also examined the health professionals’ (physicians and nurses) records in electronic clinical history regarding the recommendations of two evidence-based obesity guidelines (European and American Guidelines) [20,21] during the previous five years and whether or not records were kept in the clinical history and followed up on. Both international evidence-based guidelines were in agreement on how the management of obesity in primary care should be performed. Their key recommendations were comprehensive lifestyle programs that include a reduced calorie intake, increased physical activity, and measures to support behavioral change. The definition of meeting therapeutic guidelines was the existence of any (a, or b, or c, or d, or e) record (of each of the following prescriptions: 1. Diet, 2. Physical activity or 3. Behavioral counseling) in the clinical history during the study period regarding 1. diet ((a) the prescription of diet, specifying (b) the type of prescribed diet (hypocaloric diet) and (c) the follow-up of the diet in medical appointments), 2. physical activity ((a) the prescription of physical activity, identifying (b) the type of physical activity, (c) the duration, frequency, intensity (d) increasing and (e) follow-up), and 3. behavioral counseling ((a) the prescription of behavioral change activities, indicating (b) the type of prescription and (c) follow-up). Behavioral counseling addresses behaviors that require a change in order to achieve successful weight loss such as self-monitoring food intake (e.g., dietary record), physical activity, and monitoring body weight. Behavioral counselling includes providing advice and techniques on controlling the process of eating; slowing the rate of eating; decreasing the size of food portions, avoiding snacking between meals, not skipping breakfast, avoiding eating at night time, managing and reducing episodes of a loss of control or binge eating, practicing stimulus control and reinforcement as well as relaxation techniques. We also analyzed and collected data on the number of clinical histories of patients that had records that met all recommendations (a, b, c, etc.) on diet, physical activity or of behavioral change. The main measures were the record in the clinical history of diet prescription, physical activity, and behavioral therapy, a follow-up in consultation and compliance with the obesity guidelines recommendations.

The study was conducted under the standards and ethical criteria established in the latest Declaration of Helsinki (Fortaleza, Brazil) and were approved by the Clinical Research Ethics Committee at the Guadalajara University Hospital (CEIC_HUG_29_06_12). All participants were asked for informed consent.

For the statistical analysis, the SPSS v 23.0 program was used (IBM, ‎Armonk, NY, USA). For the comparison of means, the Student t-test was used when the variables presented normal distribution, and the Mann–Whitney U test and Wilcoxon tests were used if the variables were not normally distributed. For the comparison of proportions, the Pearson chi-square test/Fisher’s exact statistic was used. The comparison of prevalence at the beginning and end of the study was carried out using the Mc Nemar test. Multivariate linear regression analysis was performed to examine the association of the variables studied with BMI and waist circumference. Two-side *p* values < 0.05 were considered significant.

## 3. Results

### 3.1. Characteristics of the Participants

The general characteristics of the participants are shown in Table 1.

A total of 57.9% of the participants were female and the average age was 65.8 ± 12.7 years. A small reduction in BMI was observed throughout the study period (34.6 ± 4.2 vs. 34.2 ± 4.4 kg/m^2^, *p* = 0.037), while the WC increased by 4.4 cm (107.0 ± 9.9 vs. 111.4 ± 12.0, *p* < 0001) (Table 2).

The prevalence of cardiovascular risk factors increased significantly except for smoking during the study period. There was a significant increase in the prevalence of acute myocardial infarction (6.7% vs. 10.5%, *p* = 0.008) and obstructive sleep apnea syndrome (3.3% vs. 9.6%, *p* < 0.0001) (Table 2).

### 3.2. Treatment Recorded in Clinical History in Relation to the Recommendations of Obesity Guidelines and Patient Adherence to Treatment

A total of 79.9% of the electronic clinical histories recorded diet prescription, 88.5% advised physical activity and 2.9% recommended behavioral change. With respect to follow-up consultations with a health professional, 69.9% of the records recorded a following-up on diet, 76.6% on physical activity, and 1.9% on behavioral change (Table 3).

Drug treatment was indicated in 96.2% of the patients, as recommended in the obesity guidelines, but medication was only prescribed in 1.4% (Table 3). The hypocaloric diet was only registered in 28.2% of the clinical histories.

The type of physical activity was recorded in 82.8% of the patients’ histories. The time, intensity, and frequency were detailed in 64.1%. However, only 1.9% specified that there had to be an increase in baseline physical activity. Some type of behavioral change advice was registered in 1.5% of the clinical histories (Table 3).

Only 25.4% of clinical records met all the criteria established in the therapeutic guidelines regarding diet prescription. This was significantly higher for males than for females (36.4% vs. 17.4%, *p* = 0.002). A total of 1.4% met the criteria in terms of physical activity and 1.5% in relation to behavior change (Table 3).

With respect to the adherence of patients to treatment, only 12.4% reported following the advice on diet, and this was significantly higher in males than females. A total of 23.4% of patients carried out physical activity in line with the recommendations given in the health center and 0.5% followed recommendations on behavioral change (Table 3).

**Figure 1:**
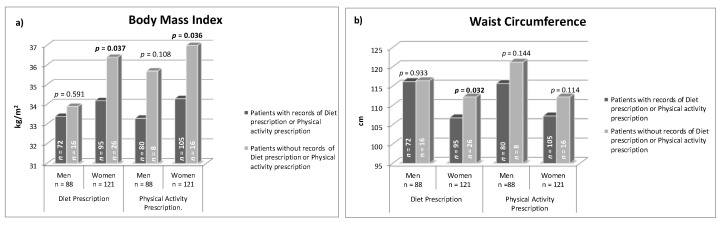
(**a**) Current mean body mass index and (**b**) mean waist circumference in patients with and without records of diet or physical activity prescription, in clinical history.

**Figure 2:**
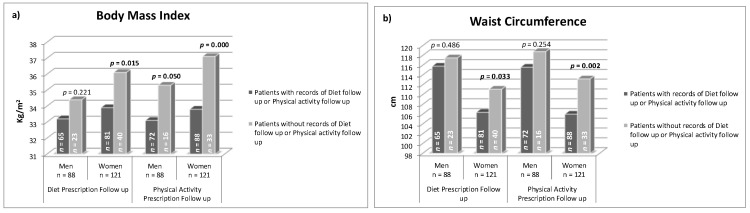
(**a**) Current mean body mass index and (**b**) mean waist circumference in patients with and without records of diet or physical activity follow-up, in clinical history.

The patients whose records mentioned diet prescription had lower average BMI and WC. Similarly, the patients with records of prescribed physical activity showed lower BMI and WC figures (Figure 1). Moreover, the patients who received follow-up consultations for both factors (diet and physical activity) had lower average BMI and WC (Figure 2). This occurred mainly in women. Although, in the multivariate analysis (Table 4), adjusted by baseline BMI and WC, the association was not significant.

## 4. Discussion

Among the findings of our study, it is worth noting that records in clinical electronic history showed full compliance to recommended decision algorithms regarding diet prescription, physical activity, and behavioral change, in only 25.4%, 1.4% and 1.5% of the clinical histories, respectively. Moreover, the patients whose records mentioned diet prescription and physical activity and who received follow-up consultations for both factors had lower average BMI and WC, but this was not significant after adjusting for baseline. Records in clinical histories could show a greater interaction with patients that improves weight loss and reduces the patient’s adiposity. Although, this could also mean that those with lower baseline BMI and WC were more likely to receive dietary and PA prescription and follow up.

The electronic health records of a cohort study of British adults on therapeutic interventions in patients with obesity in primary care revealed that only 19.8% of women and 15.8% of men with non-severe obesity had intervention during the seven-year study period [17]. In our study, we found higher prescription figures for diet and physical activity (79.9% and 88.5%, respectively); however, the advice given to patients did not meet all the recommendations included in the guidelines in terms of specifying the type of advice and follow-up. Our study identified a smaller proportion of patients receiving drug prescription (1.4%), similar for males and females. These findings coincide with the British study [17] that observed only 2.5% of men and 7.8% of women received anti-obesity drugs. As in the aforementioned study, our results suggest that the prescription of anti-obesity drugs remains low. These findings are consistent with the results of other studies that report limited prescription of weight loss medications [22,23]. Furthermore, the mean patient age of the British study was 56 years; therefore, the patients were younger than in our sample. In this sense, Patterson et al., observed that younger females were more likely to be prescribed anti-obesity medication [22]. This could suggest the influence of patient demand in the prescription of obesity treatment. While anti-obesity drugs may provide benefits, it seems that their use has been limited due to safety concerns, low effectiveness, and even a lack of knowledge among professionals of what drugs are available and their indications. It is expected that the new anti-obesity drugs will improve these results in the coming years [24].

We are aware that data obtained from electronic records may differ from conversations held with the patient and there could be a poor record-keeping of the advice provided. Regular and systematic documentation is recommended for monitoring the evolution of obesity and/or response to treatment. Their omission from the clinical record has implications for the care provided to patients, their clinical evolution, and research [12]. Technological aids, such as electronic reminders to prompt BMI measurements and prescription, in electronic record programs could improve obesity documentation and patient follow-up in consultations. To our knowledge, this study is the first to identify the relationship between recording dietetic prescription and physical exercise in the patient’s clinical record and better control of obesity.

In other studies, such as EURIKA (European Study of Cardiovascular Risk), the prescription of specific medical advice for hypertension, dyslipidemia, diabetes, obesity, and smoking wasascertained by a questionnaire addressed to the physician. These studies concluded that healthy diet advice was provided to 89.9% of patients, but only 57.8% of them were given written dietary advice. Appropriate advice on physical activity was given to 80% of patients [25]. These results coincide with ours, although their data were obtained from doctors via a questionnaire and our data source were electronic medical records. However, it is possible that the doctors who voluntarily participated in that study were more aware of achieving treatment goals.

One of the barriers that could prevent health professionals from fully aligning their clinical practice with current guideline recommendations would be the lack of training in obesity and counseling skills. A retrospective analysis of clinical records from adult patients with obesity in three primary care clinics assessed the degree of adherence of health professionals to therapeutic guidelines for obesity before and after an educational session on obesity management in primary care centers [26]. The results show that, after this educational session, there was an increase in recording dietary advice, physical activity, and behavioral change in clinical records. Additional barriers include limited time in consultations, a lack of confidence regarding the potential success of weight loss attempts, a shortage of resources, and an absence of institutional support or fear of a negative reaction from patients [12,27].

Concerning the adherence of patients to the prescribed treatment for obesity, we found that only 12.4% of patients indicated carrying out advice on diet and 23.4% of the patients reported carrying out physical activity. These results are poorer than those observed in the NHANES (National Health AND Nutrition EXAMINATION SURVEY) survey, in which 62% of patients with obesity changed their diet and 36% carried out physical activity during the 12 months preceding the study [23]. Therefore, this study suggests that engaging in exercise was less popular than dietary changes among this population, which is opposed to our findings. Although the patients in the NHANES study had a slightly higher BMI than ours (35.7 kg/m^2^ vs. 34.2 kg/m^2^), they were also younger (47.8 years vs. 65.8 years) and this could explain their greater adherence to treatment. Poor adherence to lifestyle changes may be due to unawareness, resistance to change, or mistrust of the usefulness of weight control efforts. To improve patients’ adherence to obesity treatment, primary care professionals must be involved in the treatment and follow-up of patients with obesity [28,29].

In general, we found that the change in the patients’ clinical status was unfavorable over the period studied. While BMI decreased slightly (0.4 kg/m^2^), WC increased by 4.4 cm, and comorbidities related to obesity (cardiovascular risk factors, AMI and OSAS) also increased significantly. In a longitudinal retrospective study conducted in the UK that analyzed obesity records in primary care electronic clinical histories over 10-year periods, the authors observed initial BMI figures similar to ours (34.3 kg/m^2^ in males and 35.7 kg/m^2^ in women), which increased by 1.2 and 1.3 kg/m^2^, respectively, over the period studied [30]. The higher increase in BMI in the aforementioned study could be due to a longer study period, twice that of ours, and a lower mean age at the start of the study (44 years in men and 42 years in women). Regarding the increase in WC observed in our study, this coincides with data from the NHANES survey, in which the authors observed that although the prevalence of obesity seemed to have stabilized, the average abdominal circumference among adults in the United States had continued to increase (3 cm) from 2000 to 2012 [31]. The observed increase in WC could reflect changes in body fat distribution, suggesting increases in visceral fat. This may be attributable to many factors, such as increasing age, the use of certain pharmaceuticals or even endocrine disruptors [32].

This study has some strengths and limitations. The main strength is that the data was collected at the time of the study, all the patients were interviewed and the anthropometric measurements for all the patients were taken, as opposed to being self-reported. In addition, we were able to confirm all the data recorded in the electronic clinical history, thus assessing the real situation of each patient.

The main limitations of this study is the inclusion of a single primary care health center, which may restrict the generalizability of our results from one population to another. Furthermore, the data collected from the electronic clinical records may show a low registration of some variables given that the evaluation of the implementation of obesity guidelines by health professionals was based on electronic records and not by asking health professionals directly about adherence to the guidelines. Professionals are usually very busy and may not adequately document what is prescribed to their patients. Nonetheless, a self-reported validated questionnaire would also cause some recall bias. It is likely that not all prescriptions, particularly lifestyle advice, were recorded in the electronic clinical history, which could have led to an underestimation of prescription rates. However, this is less likely to be an issue with drug prescribing. A further limitation is the average age of the population studied, which was 65.7 ± 12.7 years, so it would be interesting to extend the study to a more diversified sample in terms of age. Likewise, the period studied was five years, which could be extended to evaluate changes in the longer-term variables. Finally, due to the study’s design, no causal relationships can be extracted from the associations observed. A prospective design with interviews with health professionals would have provided a more direct reflection of the implementation of obesity guidelines. Moreover, future research to monitor patient body fat distribution, explore causes of increased WC, and analyze the efficacy of new anti-obesity medications seems warranted. However, the work provides useful information to be able to investigate more possible causes of a lack of success in treating obesity.

## 5. Conclusions

We have observed low documentation in electronic records relating to the recommendations of the evidence-based guidelines in obesity treatment. Records of dietetic and physical exercise prescription and follow-up in the clinical histories were associated with better control of obesity, but this was not significant after adjusting for baseline.

## Figures and Tables

**Table 1:** Sociodemographic, clinical and laboratory characteristics of patients affected by obesity in primary care (current data).

	Total *n* = 209	Men *n* = 88 (42.1)	Women *n* = 121 (57.9)	*p*
Age (years) Mean ± SD	65.8 ± 12.7	63.2 ± 13.6	67.6 ± 11.8	0.015
BMI (kg/m^2^) Mean ± SD	34.2 ± 4.5	33.5 ± 4.1	34.7 ± 4.7	0.059
WC (cm) Mean ± SD	111.4 ± 11.6	116.2 ± 10.1	107.9 ± 11.5	<0.0001
HBP *n* (%)	179 (85.6)	79 (89.8)	100 (82.6)	0.166
T2DM *n* (%)	81 (38.8)	34 (38.6)	47 (38.8)	1
HC *n* (%)	130 (62.2)	55 (62.5)	75 (62.0)	1
HTG *n* (%)	66 (31.7)	36 (41.4)	30 (24.8)	0.009
Smoker *n* (%)	27 (12.9)	18 (20.5)	9 (7.4)	0.007

SD, Standard deviation; BMI, body mass index; WC, waist circumference; HBP, high blood pressure; T2DM, type 2 diabetes mellitus; HC, hypercholesterolemia; HTG, hypertriglyceridemia.

**Table 2:** Change in patients’ clinical status over studied time (five years).

	5 Years before				Current				5 Years before vs. Current
	Total N = 209	Men *n* (%) 88 (42.1)	Women *n* (%) 121 (57.9)	*p* Men vs. Women	Total N = 209	Men *n* (%) 88 (42.1)	Women *n* (%) 121 (57.9)	*p* Men vs. Women	*p* Total Group
Age (years) Mean ± SD	-	-	-	-	65.8 ± 12.7	63.2 ± 13.6	67.6 ± 11.8	0.015	-
BMI kg/m^2^ Mean ± SD	34.6 ± 4.2	33.7 ± 3.8	35.2 ± 4.3	0.015	34.2 ± 4.5	33.5 ± 4.1	34.7 ± 4.7	0.059	0.037
WC cm Mean ± SD	107.0 ± 9.9	110.6 ± 8.6	104.7 ± 10.2	<0.0001	111.4 ± 11.6	116.2 ± 10.1	107.9 ± 11.5	<0.0001	<0.0001
HBP *n* (%)	161 (77.0)	70 (79.5)	91 (75.2)	0.508	179 (85.6)	79 (89.8)	100 (82.6)	0.166	<0.0001
T2DM *n* (%)	57 (27.3)	25 (28.4)	32 (26.4)	0.756	81 (38.8)	34 (38.6)	47 (38.8)	1	<0.0001
HC *n* (%)	94 (45.0)	39 (44.3)	55 (45.5)	0.889	130 (62.2)	55 (62.5)	75 (62.0)	1	<0.0001
HTG *n* (%)	57 (30.5)	31 (40.8)	26 (23.4)	0.015	66 (31.7)	36 (41.4)	30 (24.8)	0.015	<0.0001
Smoker *n* (%)	35 (16.7)	24 (27.3)	11 (9.1)	0.001	27 (12.9)	18 (20.5)	9 (7.4)	0.007	0.021
AMI *n* (%)	14 (6.7)	12 (13.6)	2 (1.7)	0.001	22 (10.5)	16 (18.2)	6 (5.0)	0.003	0.008
OSAS *n* (%)	7 (3.3)	4 (4.5)	3 (2.5)	0.458	20 (9.6)	11 (12.5)	9 (7.4)	0.241	<0.0001

SD, Standard deviation; BMI, body mass index; WC, waist circumference; HBP, high blood pressure; T2DM, type 2 diabetes mellitus; HC, hypercholesterolemia; HTG, hypertriglyceridemia; AMI: Acute myocardial infarction; OSAS, obstructive sleep apnea syndrome.

**Table 3:** Treatment recorded in clinical history in relation to the recommendations of obesity guidelines and patients’ adherence to treatment.

	Total (*n* = 209)	Men (*n* = 88)	Women (*n* = 121)	*p*
**Indication of obesity drug *n* (%)**	201 (96.2)	85 (96.6)	116 (95.9)	1.000
Prescription of Obesity drugs recorded *n* (%)	3 (1.4)	1 (1.1)	2 (1.7)	1.000
**(1) Diet**				
(a) Prescription of diet recorded *n* (%)	167 (79.9)	72 (81.8)	95 (78.5)	0.603
(b)Type of prescribed diet: Hypocaloric diet *n* (%)	59 (28.2)	34 (38.6)	25 (20.7)	0.005
(c) Recorded follow up *n* (%)	146 (69.9)	65 (73.9)	81 (66.9)	0.291
Clinical histories of patients that had records that met all recommendations on (a) prescription of diet, (b) type of diet and (c) follow up	53 (25.4)	32 (36.4)	21 (17.4)	0.002
**(2) Physical activity**				
(a) Prescription of physical activity recorded *n* (%)	185 (88.5)	80 (90.9)	105 (86.8)	0.389
(b) Type of physical activity (e.g., walking, running, etc.) recorded *n* (%)	173 (82.8)	73 (83.0)	100 (82.6)	1.000
(c) Duration, frequency and intensity of physical activity recorded *n* (%)	134 (64.1)	58 (65.9)	76 (62.8)	0.664
(d) Increasing physical activity recorded *n* (%)	4 (1.9)	2 (2.3)	2 (1.7)	1.000
(e) Recorded follow up *n* (%)	160 (76.6)	72 (81.8)	88 (72.7)	0.139
Clinical histories of patients that had records that met all recommendations on (a) prescription of physical activity, (b) type of physical activity (c) duration, frequency and intensity, (d) increasing physical activity and (e) follow up	3 (1.4)	1 (1.1)	2 (1.7)	1.000
**(3) Behavior change**				
(a) Prescription of behavior change recorded *n* (%)	6 (2.9)	3 (3.4)	3 (2.5)	0.698
(b) Type of behavior change recorded *n* (%)	2 (1.5)	1 (1.8)	1 (1.3)	1.000
(c) Recorded follow up *n* (%)	4 (1.9)	2 (2.3)	2 (1.7)	1.000
Clinical histories of patients that had records that met all recommendations on (a) Prescription of behavior change, (b) type of behavior change and (c) follow up	2 (1.5)	1 (1.8)	1 (1.3)	1.000
**Patients adherence to treatment**				
Patients adherence to the prescribed diet *n* (%)	26 (12.4)	17 (19.3)	9 (7.4)	0.018
Patients adherence to the prescribed physical activity *n* (%)	49 (23.4) (49)	23 (26.1)	26 (21.5)	0.509
Patients adherence to the prescribed behavior change *n* (%)	1 (0.5)	0 (0.0)	1 (0.8)	1.000

**Table 4:** Lineal regression model (women): (**A**) BMI (dependent variable); (**B**) WC (dependent variable).

(A) BMI	Standardized ß	*p*	CI 95%
Prescription of diet recorded (79.9%)	0.089	0.400	−1.373/3.411
Follow up diet recorded (69.9%)	−0.028	0.813	−2.607/2.049
Prescription of PA recorded (88.5%)	−0.121	0.196	−4.229/0.875
Follow up PA (76.6%)	0.172	0.119	−0.474/4.105
Age	−0.115	0.086	−0.099/0.007
**(B) WC**	**Standardized ß**	***p***	**CI 95%**
Prescription of diet recorded (79.9%)	0.066	0.656	−8.437/13.322
Follow up diet recorded (69.9%)	−0.250	0.163	−18.265/3.131
Prescription of PA recorded (88.5%)	0.132	0.263	−5.173/18.630
Follow up PA recorded (76.6%)	0.238	0.192	−3.975/19.400
Age	−0.068	0.491	−0.291/0.141

BMI, Body mass index; WC, Waist circumference; PA, Physical activity. (**A**) Model adjusted (by baseline BMI) R^2^ = 0.567. (**B**) Model adjusted (by baseline WC) R^2^ = 0.366.
